# Supplementary material for: Dreaming of the sleep lab
Source: PLoS One. 2021 Oct 6;16(10):e0257738. doi: 10.1371/journal.pone.0257738 (PMC8494361; doi:10.1371/journal.pone.0257738)
Supplement: S1 Appendix — (PDF) [file pone.0257738.s001.pdf]

Scoring of Laboratory Incorporation Dreams (SoLID) Criteria

Instructions:

The SoLID Criteria were designed to assess the multiple ways that participants report dreaming about the sleep lab. They are intended to be comprehensive criteria, including both direct and indirect references to the lab as evaluated in previous studies, but steering clear of purely metaphorical references. They also include subjective sensory impressions and feelings of self-consciousness and performance anxiety that are commonly reported by laboratory participants. However, the criteria are not designed to assess the complete range of emotional reactions possible in a sleep lab experiment.

Step 1 of the procedure is for a judge very familiar with the sleep lab and its procedures to screen all candidate dream reports for whether or not there is any trace of the laboratory in the report. Such traces include lab-related people, places, objects, tasks and sleep activities (categories 1-5). If any are identified, the dream is coded as '1'. If not, the dream is coded as '0' on all categories and it is given no further consideration.

Step 2 involves scoring the dream report on the 14 specific and 1 global categories.

- The 14 specific categories all use a binary 0/1 score where 0 means the feature is *absent* and 1 means the feature is *present*.
- The global category is scored on a 1-7 scale where 1=*mild indirect reference to the lab experience*, and 7=*very clear and extensive incorporation of multiple aspects of the lab experience*.

Scoring criteria and examples are provided in the Table below.

Category	Criteria	Examples
**Lab elements**		
1. People	Experimenter (or someone in E role), hospital personnel, other participants	*The assistant woke me up; My mom was in a lab coat; A nurse was taking my blood pressure*
2. Places	Lab, hospital, bedroom, adjacent hallways, lab bathroom, 'here'. 'Places' is not scored if the report only mentions 'in this study'	*An assistant came into the bedroom; I was in a hospital*
3. Objects	Any objects related to lab or experiment: electrodes, gel, pillow, cameras in bedroom, computers and other equipment	*I still had electrodes on my head; I was looking at my brain waves on the computer screen*
4. Tasks	Any reference to being part of a study, including being evaluated by tasks, questionnaires, sleep recordings, dream reporting and other experimental procedures	*I was in this study; I was being examined on a new task; I had to report my dreams*
5. Sleep Activities	Any reference to sleep, sleep activities, or sleep-related states (fainting, fatigue), including bedroom, bed, pajamas—in self or others— happening outside the lab	*I woke from a nap at home; My brother was wearing pajamas; I suddenly felt very tired and tried to find a place to sleep*
**Lab themes**		
6. Sleep Performance	Any concern that their sleep in the lab is	*I was worried I would fail the dream test; My sleep was too*

		inadequate or in question	*short; I was afraid I couldn't fall asleep*
7.	Wayfinding	Participant actively ambulates in, around, into or out of the lab or hospital. Passive movement is not scored	*I was trying to find my way back to the lab; I got lost in the hospital hallways*
8.	Sensory	Real sensations occurring during sleep in the lab, including muscle atonia, interoception (e.g., hunger, thirst, having an urge to urinate), somatosensory perception (touch, pain, temperature), and auditory/visual/tactile related to specific stimuli that are part of the experiment; excluding emotions	*I felt electrode paste in my hair; I needed to pee very badly; I couldn't use my right arm; My feet were cold*
9.	Meta-dreaming	Any alteration of consciousness: lucid, pre-lucid, questioning sleep, false awakening/false waking, sleep paralysis, out of body experience	*I wondered if I was really sleeping; I realized I was dreaming; The dream in my dream…*
10.	Object of observation	Lab-related act or feeling of being observed, evaluated, criticized or praised by others about one's sleep/dreams or measures thereof; feelings of intimacy or breach of it, loss of control, including aggressive or violent acts toward the self	*There was a big window between the control room and bedroom; Some person was assigned to sleep in the bed with me; My dreams were being transcribed directly on paper*
11.	Friends/Family in lab	Family member, friend or acquaintance participating in the experiment or simply being present in the lab	*My parents were doing the experiment with me; My friend was sleeping in one of the lab bedrooms*
**Temporal orientation**			
12.	Past	Lab-related events taking place in the past	*I was being hooked up to electrodes; I was heading to the lab*
13.	Immediate Future	Lab-related events taking place just after the sleep period is over or after last awakening, including awakening in, or being awakened in, the lab	*You woke me for a dream report; It was already noon and the study went on for too long*
14.	Future	Any post-lab activities if continuous from (related to) the lab	*We took the metro home after the study; I was telling my friends about the experiment I just did*
15.	Global	Overall impression that the dream was influenced by the lab; overall richness of lab incorporations (1-7 scale)	*See Table 2 for example of dream with maximum Global score*
