# Supplementary material for: Dreaming of the sleep lab
Source: PLoS One. 2021 Oct 6;16(10):e0257738. doi: 10.1371/journal.pone.0257738 (PMC8494361; doi:10.1371/journal.pone.0257738)
Supplement: S2 Appendix — (PDF) [file pone.0257738.s002.pdf]

Exemplary lab incorporation dreams

The following exemplary dream reports are all from REM sleep nap awakenings. Time of awakening, age and sex of participants, and original language of the dream report are shown in brackets for each example.

Dream example #1

[11:23 am, 22 yrs old, F, Original report in English]

I was dreaming that I was undergoing the study, actually. I dreamed that **it was already noon**, and this very **study had gone on longer than anticipated** because **I wasn't able to fall asleep**. Two male researchers were at a disagreement about whether allowing me to continue would be worth the time. So I was **trying to fall asleep and not doing very well**, not withstanding because **there was no real barrier** between **this bed and the researchers, no door**, just a closet for coats and then the TVs around the corner, because there was music playing in the background one of the student researchers was mock-offended that I didn't share her taste in music and - at one point - that my bed was in fact outside, supposedly for the ambience but was surrounded by the bike path [near the hospital], with cyclists occasionally **weaving in circles around my bed**. The researchers had **referred to my brain patterns** during my attempted-nap as a "Dante pattern", which is either indicative of multiple personalities while awake or weaving in and out of sleep while attempting to sleep. Just before actually waking up, **I was trying to fall asleep in my dream** by holding onto an office chair and rocking it back and forth, **aware of the cameras** but **desperate enough to fall asleep** that I didn't really care. I was also dreaming of the **mental exercises** that would usually help me slow down my thoughts enough to fall asleep except they **kept being corrupted**, so instead of fields and beaches it was buildings and people. So this part of my dream was layered **between my dream-self and the mental experiences of my dream-self**.

//

Dream example #2

[11:09 am, 23 yrs old, F, Original report in English]

I dreamt about this very room, **like my dream had been hijacked**. I heard a strange voice say something death, died, dream something in this, something to the extent of that. I thought maybe you were playing a spooky soundtrack **in order to influence my dreams**. I **got lost wandering** through the hospital, and found myself outside with all the **gear still stuck to my face**. Wandering through all the corridors **trying to remember my way back**. The sun was excessively bright. I dreamt about **seeing myself in this room**. It was hard to tell if I was dreaming or not.

//

Dream example #3

[12:43 pm, 22 yrs old, M, Original report in French]

I'm in the bedroom in the lab, where I was actually sleeping. The TV which is to my left in reality is found on my right in my dream. I use it to listen to soundtracks to **help me fall asleep**. […dreamer then watches an episode of The Office in the dream…]. I turn off the TV, and I tell

myself that I **have to fall asleep to have a dream for the study in which I am participating**. I am not moving, and I start to feel **vibrations in my whole body**. I also feel like I'm **starting to levitate in the air**. I'm excited, as this is what usually happens to me when I consciously fall asleep, so I'm glad I could **lucid dream** while participating in the study. Because of my anticipation, I couldn't go into a dream (in my dream). A few moments later, [the experimenter] **comes into the bedroom** and tells me that I can get up, that **the time for sleeping is over**. I'm not sure if it's a good idea to move, because I might still be able to lucidly fall asleep. [The experimenter] leaves the room, and I see in front of me, **where there should be a wall, a door**. The door is opened by Jim, another character from The Office. I see on the other side of the door **a small corridor**, and less than a yard in front of the open door, there is **another open door** which gives the view on a small dining room with a fridge. Jim tells me I can get up. I ask him if it's possible for me to **go and eat**, and he tells me yes (I **hadn't eaten all day**).

//

Dream example #4

[11:12 am, 26 yrs old, M, Original report in French]

I thought I had no dream, because I **dreamed of the laboratory** and specifically of the bedroom, **and the procedure to follow if the dream is lucid** (moving my eyes from left to right). The first memory of my dream starts with this procedure, after doing it the **researcher came slowly into the room**, turned on the light and **gave me directions** then another person came to talk to me, she cared for **conscious people in dreams**. From that moment I realized that the room had **another door with a small corridor** where there were two people in **white uniform**s who were cleaning medical instruments at the end of this corridor in a room with the door closed. When **the door opens** a gentleman met my gaze but I had the impression that he shouldn't have, as if he had something to hide. I could see from my bed, some details, a desk and a computer. This man was tall, broad like Bruce Willis, dressed in a sky blue shirt and an FBI colored jacket. Then **I thought I was awake** and **waiting for the end of the experiment**, **I glued one of my electrodes back on**.

//

Dream example #5

[11:10 am, 22 yrs old, F, Original report in French]

I dreamed of **waking up here in the lab**. I woke up and [the experimenter] explained to me that **it was over and I could go home**. Eventually, I'm not sure why, but you were going to **drive me to a bus** and we would even take the bus all 3 together and at the same time you would start **removing the electrodes** from me. Then, at one point I point out that we did not redo the second virtual reality session and then we realize that we did nothing of all the rest that had to be done after the nap. So we go back. There are other people this time, another participant. And then I really want to **go to the bathroom** so I go to the small room next door and I can't quite close the blinds, **there is a window** that overlooks the small room where the toilet is. It's a room with a bed too. Anyway, it's complicated but I ended up succeeding and then I think I also **woke up halfway in my room** because I found myself in bed but it's hard to say if I was dreaming about this too or not. I also remember a scene where **I tell [the experimenter] that I haven't slept so much and hope it's not too bad** but she tells me that I actually slept well all along

and then we **look a little at my data**. But again I think I woke up often so my dreams are interrupted.

//

Dream example #6

[12:38 pm, 30 yrs old, M, Original report in English]

I woke up due to **pain in my arm**, which I thought was **coming from the electrodes**. I left the room to find [the experimenter] or someone else, but no one I recognized was around. I **walked into the hallway** and found many children with disabilities playing there, supervised by adults. They looked at me kind of funny and I remembered that I had **wires attached to my face**. I asked a girl if she knew where the people conducting the dream study might have gone, but she didn't know. She seemed interested in the study, explaining that **she had participated in several studies**, including one where **they made incisions on her back and took grafts of skin**. A 17-year-old (I'm guessing age) with some sort of disability laid down on the floor in front of me and grabbed me by the ankles. […]. Upon returning to the room, I found all these other people in there, but none of the people who were working with me. Everyone was **crowded around the computer screens**. They looked like supervisors or **interested researchers**. I went back in the room that I was sleeping in and **found another doorway** off the far wall that lead to **another room the size of my bed**. A couple **people from my yoga class** walk in there and **go lie on the bed**. I came back into the room where I was sleeping and there were maybe two people sleeping the bed I was supposed to be in, plus two cots set up with people in them. I told the people that maybe **they should not fall asleep in this room because I hadn't finished my study.** They didn't listen or respond. I was slightly annoyed, but mostly because **I still felt sleepy and wanted to nap some more**. I walked back into the lab and looked at the time on a digital clock with red numbers. **It was 3:30pm**.

//

Dream example #7

[12:04 pm, 23 yrs old, F, Original report in English]

I was in a place sort of **like this room**, but it had **large windows**. In my dream, I was in a **semi-conscious state**, and there was a male character, who seemed to be like a research nurse or something. While I was still lying in a bed, someone took me out on **a ride through the town** in the back of a vehicle like an ambulance, but **with windows**. I was very confused in the dream, as the scenes and setting kept on changing, between the room and the vehicle. I also heard people talking and was **not sure if they were trying to trick me as to where I actually was**. My left **foot felt cold**, but I think that is because this room is quite cold.

//

Dream example #8

[11:10 am, 26 yrs old, F, Original report in French]

I dreamed that the **experiment was going on too long**, that **I didn't sleep enough**, that **it was too long**. I felt like maybe you **([the experimenter]) were disappointed in me** or that I **wasn't 'performing' well enough** on the sleep side. I think it's the feeling I had not long before waking

up, or the beeps waking me up: I was also confused and I wasn't sure what woke me up. […] I remember thinking several times, in my dreams, of you too, of wondering if you could possibly **see my dreams through the electrodes** and **if you were going to find me weird**.

//

Dream example #9

[11:18 am, 33 yrs old, F, Original report in French]

I was in the lab doing the dream tests. **My mother was with me**, we walked together **in a corridor** that brought us into the testing room. There were **several busy people**. My mom called the person who was going to do the test, I told her, wait, because she's busy. After that I did a test where she put **electrolytes in my head** and it was supposed to **write the dreams directly on the paper**. I finished the test and **forgot to write the dream** on paper. I went to **another corridor** to do the second test. My mother had a loaf in her hand, the size of a baguette. When we arrived in the room, the person asked me if I gave my CV as well. I told them no, I don't understand what's the point of leaving my CV with the dream. **My sister was also with me and my mother** at the time. We were laughing, we were happy and we wanted to finish the test to **get out of the laboratory**. The person said we could start and I remember **knowing that I was dreaming** and **worrying about waking up and forgetting the dream**.
